# Hip and groin injury is the most common non-time-loss injury in female amateur football

**DOI:** 10.1007/s00167-018-4996-1

**Published:** 2018-06-02

**Authors:** Rob Langhout, Adam Weir, Wendy Litjes, Maarten Gozeling, Janine H. Stubbe, Gino Kerkhoffs, Igor Tak

**Affiliations:** 1 Department for Manual Therapy and Sports Rehabilitation, Physiotherapy Dukenburg Nijmegen, Aldenhof 7003, 6537 DZ Nijmegen, The Netherlands; 2 Amsterdam Collaboration for Health and Safety in Sports (ACHSS), AMC-VUmc IOC Research Center, Amsterdam, The Netherlands; 3 Academic Center for Evidence-Based Sports Medicine (ACES), Amsterdam, The Netherlands; 4 Advanced Studies Manual Therapy, SOMT University, Amersfoort, The Netherlands; 5 Dutch Center for Allied Health Care (NPi), Amersfoort, The Netherlands; 6 Sports Groin Pain Centre, Aspetar Orthopaedic and Sports Medicine Hospital, Doha, Qatar; 7 Department of Orthopaedics, Erasmus MC Center for Groin Injuries, Erasmus MC University Medical Centre, Rotterdam, The Netherlands; 8 Department of Sports Rehabilitation, Physiotherapy Wijchen, Wijchen, The Netherlands; 9 Department of Physiotherapy, PSV Eindhoven, Eindhoven, The Netherlands; 10 Codarts Rotterdam, University of the Arts, Rotterdam, The Netherlands; 11 Department of Orthopaedic Surgery, Academic Medical Center, University of Amsterdam, Amsterdam Movement Sciences, Amsterdam, The Netherlands; 12 Department for Manual Therapy and Sports Rehabilitation, Physiotherapy Utrecht Oost, Utrecht, The Netherlands

**Keywords:** Female football (soccer), Female athlete, Groin pain, Hip and groin injury

## Abstract

**Purpose:**

Hip and groin injuries in football are problematic due to their high incidence and risk of chronicity and recurrence. The use of only time-loss injury definitions may underestimate the burden of hip and groin injuries. Little is known about hip and groin injury epidemiology in female football. The first aim of this study was to examine the within-season (2014–2015) prevalence of total injury with and without time-loss in female amateur football players. The second aim was to study the within-season and preseason (2015–2016) prevalence of hip/groin injuries with and without time-loss. The third aim was to study the association between the duration of hip and groin injury in the 2014–2015 season and the severity of hip/groin problems during the 2015–2016 preseason.

**Methods:**

During the preseason, 434 Dutch female amateur football players completed an online questionnaire based on the previous season and current preseason. The hip and groin outcome score (HAGOS) was used to assess the severity of hip and groin injuries.

**Results:**

The hip/groin (17%), knee (14%), and ankle (12%) were the most frequent non-time-loss injury locations. The ankle (22%), knee (18%), hamstring (11%), thigh (10%), and hip/groin (9%) were the most common time-loss injury locations. The previous season prevalence of total injury was 93%, of which non-time-loss injury was 63% and time-loss injury was 37%. The prevalence of hip/groin injury was 40%, non-time-loss hip/groin injury was 36% and time-loss hip/groin injury was 11%. The preseason prevalence of hip/groin injury was 27%, non-time-loss hip/groin injury was 25%, and time-loss hip/groin injury was 4%. Players with longstanding hip/groin injury (> 28 days) in the previous season had lower HAGOS scores at the next preseason than players with short-term (1–7 days) or no hip/groin injury (*p* < 0.001). From all players with hip/groin injury from the previous season, 52% also sustained hip/groin injury in the following preseason, of which 73% were recurrent and 27% were chronic hip/groin injuries.

**Conclusion:**

Injury risk, and especially non-time-loss hip and groin injury risk, is high in female amateur football. Three-quarters of the players with longstanding hip and groin injuries in the previous season have residual problems at the start of the following season.

**Level of evidence:**

II.

**Electronic supplementary material:**

The online version of this article (10.1007/s00167-018-4996-1) contains supplementary material, which is available to authorized users.

## Introduction

The number of female football players in Europe is growing rapidly, and female participation rates in the US almost equal those of males [28]. Dutch female football has increased rapidly, with 23% more players over the past 5 years and 153,001 registered players in the 2016–2017 season. It is now the largest female team sport in Holland [28].

Despite its popularity and growth, injury studies in female football lag far behind those in male football [21]. In addition, most injury or risk factor studies use only time-loss injury (TLI) definitions [7, 30]. The within-season prevalence of TLI in elite female football ranges between 38 and 48% [4, 6, 10, 11, 14, 15, 20]. Non-time-loss injury (NTLI) has been less studied in football [30]. The little available data suggest, as expected, that NTLI is more common than TLI [9, 17].

Studies reporting specifically on hip and groin injury (HGI) are hard to compare, as they use different injury terminologies and definitions [2, 32]. A study in elite female football players found that injury rates were four times higher (36 vs. 9%) for non-time-loss HGI (NTL-HGI) than for time-loss HGI (TL-HGI) [9]. A recent systematic review showed that, in elite female studies, prevalence rates of TL-HGI ranged from 2 to 11% [30]. The use of TL-HGI definitions probably underestimates the true burden of HGI [7, 26]. HGI is common in (sub-) elite male football and is known for its high incidence, chronicity, and risk of recurrence [19, 26, 34]. Injury risk and prevention has yet not been studied in female amateur football players [30].

Patient-reported outcome measures (PROs) are the gold standard for assessing the perceived health status of specific populations and injuries [16]. The hip and groin outcome score (HAGOS) is developed for young and active individuals, measures the severity of hip- and groin-related problems, and is validated in several languages, including Dutch [23, 25, 27].

Limited literature exists on female football players and especially on the hip/groin injuries. Most literature on this topic studied professional players, although the amount of amateur football players is the majority of the people that visit the sports clinic. Therefore, the first aim of this study was to examine the within-season (2014–2015) prevalence of total injury burden (NTLI and TLI) in female amateur football. The second aim was to study the within-season and preseason (2015–2016) prevalence of hip and groin injury (NTL-HGI and TL-HGI). The third aim was to examine the association between the duration of HGI in the 2014–2015 season and the severity of hip/groin problems during the 2015–2016 preseason.

## Materials and methods

In this cross-sectional survey study, female amateur football players completed an online questionnaire during the 2015–2016 preseason. The ‘Strengthening the Reporting of Observational Studies in Epidemiology’ (STROBE statement) was used to report the findings of this study [29]. By clicking the “I participate” link in the electronic questionnaire, the participants gave their consent that their anonymized data could be used for research purposes.

### Participants

All participants were female amateur players in the Dutch women’s football league, as registered by the Royal Dutch Football Association (KNVB). To obtain a large sample size, 43 teams (645 players), representing all amateur playing levels (top class, sub-top class, 1st–6th class) from all KNVB districts were selected and invited by e-mail to participate in this general injury survey. Every player received information by e-mail about the study and instructions for completing the questionnaire (Supplementary Appendix). Players were included if they were female, were between the ages of 18 and 40, and had played amateur football during the previous season, regardless of being injured or not. Professional players and those from the veteran’s leagues were excluded. The parameters of age, height, weight, weekly average exposure (training and matches), leg dominance (defined as the preferred kicking leg), and playing levels were self-reported.

### Injury registration

Time-loss injury (TLI) was defined as ‘Any physical complaint sustained by a player as a result of a football match or training, resulting in a player being unable to fully take part in future football training or match play’ [7]. Non-time-loss injury (NTLI) was defined as a situation where players experienced ‘Any physical complaint as a result of a football match or training, but without time-loss’ [7]. The same definitions applied for hip and groin injury, referring to NTL-HGI and TL-HGI. The presence of injury was scored by dichotomous answer options (yes/no).

When present, the duration (days) of both NTL-HGI and TL-HGI was noted and classified as minor (1–7 days), moderate (8–28 days), or major (> 28 days), according to the international classification for football injuries [7]. In addition, the manner of onset (maximal kicking, sprinting/running, cutting/pivoting, and other) of HGI was registered for the 2014–2015 season. An online registration system was used (Google Forms).

### Injury region

A body chart was used to illustrate all locations of NTLI and TLI based on the Dutch Injury Information System framework and Orchard Sports Injury Classification System [18]. For this study, the hip/groin was referred to as ‘the region between the front of the hip and the inner front of the thigh’ [18]. A chart of the hip and groin region was used to address the location of HGI in this region.

### Hip and groin outcome score (HAGOS)

The HAGOS was used to assess the severity of hip- and groin-related problems for all players on six subscales: pain (P), symptoms (S), activities of daily living (ADL), sport and recreation (SR), participation in physical activities (PA), and quality of life (QOL) [27]. Subscale scores range from 0 to 100, where 0 indicates severe hip and groin symptoms and problems, and 100 indicates no symptoms or problems [27]. HAGOS is available in the Dutch language and is found to be reliable (ICCs between 0.83 and 0.87), internally consistent (Cronbach’s *α* between 0.81 and 0.92), valid in young athletes (including football players), and comparable to the original Danish version [23]. The mean ± SD test–retest differences for the six subscales were 0.5 ± 10.9 (P), 1.7 ± 10.4 (S), 0.4 ± 14.2 (ADL), 2.8 ± 15.8 (SR), 2.3 ± 18.9 (PA), and 2.5 ± 11.5 (QOL).

### Survey period

Participants were asked to complete the injury questionnaire (including HAGOS) during an 8-week period in the preseason of 2015–2016 (August, September, and October 2015). NTLI and TLI were retrospectively assessed per body location for the previous season (1 August 2014–15 June 2015). NTLI-HGI and TL-HGI were assessed for the previous season and for the current preseason. History of HGI was assessed for the period prior to the 2014–2015 season. The HAGOS scores concerned the player’s health status for the week prior to completing the questionnaire (see the Supplementary Appendix for the survey and HAGOS at http://www.koos.nu).

### Bias

To minimize recall bias, dichotomous answer options, definitions of the terms used, and assisting figures that specified anatomical regions were employed [16]. Adequate reliability between retrospective and prospective dichotomous registration of self-reported injuries has been previously observed [3].

### Approval

This study complied with the requirements of the declaration of Helsinki [35]. The Dutch Central Committee on Research Involving Human Subjects (CCMO) states that no medical ethical approval was necessary for this questionnaire study. Participants were neither physically examined nor treated by any means. As such no burden existed nor were they denied any treatment. This is stated in the Dutch Medical Research Involving Human Subjects Act (WMO; http://wetten.overheid.nl/ BWBR0009408).

### Statistical analysis

The data were tested for normality using the Kolmogorov–Smirnov test. Normally distributed data are presented as a mean and standard deviation (SD). Non-normally distributed data are presented as a median and interquartile range (IQR 25–75%). The presence and locations of NTLI and TLI are presented as absolute (counts) and relative (percentage of total). To avoid overestimation, HGI was defined as the total number of players with NTL-HGI and TL-HGI minus the number of players with both injuries. The duration (days) of NTL-HGI and TL-HGI was analysed by frequencies and percentage of the total number of players. The average number of players for an average squad was calculated to examine the number of injuries per squad per season. To calculate duration (days) of NTL-HGI and TL-HGI per squad, an arbitrary duration of 3 days was chosen for minor HGI, 18 days for moderate HGI, and 28 days for major HGI, to prevent overestimation.

Match and training exposure were determined (hours) and 1 match represented 1.5 h. A Mann–Whitney *U* test was used to examine differences between HAGOS scores for HGI, no HGI, and HGI duration groups. Incorrect or missing data were reported and corrected by the means of the variables and frequencies. The level of significance was set at *α* < 0.05. The data were analysed using SPSS 23 (IBM, Armonk, USA).

## Results

**Fig. 1:**
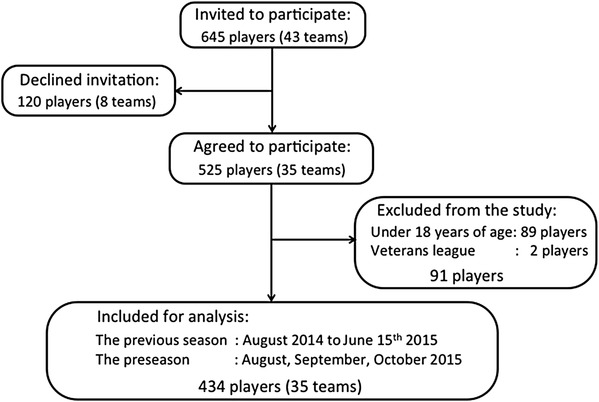
Flowchart showing player inclusion and exclusion

Of the 43 teams invited, 8 teams (120 players) declined the invitation and 35 teams participated in this study (response rate 81%). This resulted in 525 female players, from which 91 (17%) failed to meet the inclusion criteria of being at least 18 years of age (*n* = 89) or participating in the included playing levels (*n* = 2 veterans league). Data from 434 players were used for the analysis (Fig. 1).

During the previous season, the 434 players had a total exposure time of 64,034 h (50,720 training and 13,314 match hours). On average, each player spent 148 ± 58 h playing football (117 ± 54 training and 31 ± 12 match hours) during the 40-week competitive season. An average team consisted of 14.7 ± 0.7 players. Player characteristics are shown in Table 1.

**Table 1:** Player characteristics (*n* = 434)

Age (years)	24.2 (5.1; 18–52)
Height (cm)	170.7 (6.0; 155–190)
Weight (kg)	66.4 (8.7; 46–110)
Body mass index (kg/m^2^)	22.6 (2.7; 17.1–40.0)
Match exposure (total matches per season)	20.9 (8.7; 0–60)
Training exposure (hours per week)	3.0 (1.4; 0–12)
Playing level, *n* (%)	
Top class	23 (5)
Sub-top class	48 (11)
First class	60 (14)
Second class	51 (112)
Third class	35 (8)
Fourth class	95 (22)
Fifth class	89 (21)
Sixth class	33 (8)
Leg dominance, *n* (%)	
Left	45 (10)
Right	389 (90)
HAGOS subscales	
Pain (P)	100.0 (90.0–100.0)
Symptoms (S)	89.3 (78.6–100.0)
Activities of daily living (ADL)	100.0 (95.0–100.0)
Sports and recreation (SR)	100.0 (87.5–100.0)
Participation in physical activity (PA)	100.0 (75.0–100.0)
Quality of life (QOL)	100.0 (85.0–100.0)

Player characteristic presented as the mean (SD, range) or median (IQR 25–75). Exposure is presented for the previous season (2014–2015)

*y* years, *cm* centimetre, *kg* kilogram, *kg/m*^*2*^ kilogram/square metre, *IQR* interquartile range, *n* number

### Total injury during the previous season

For the previous season, 404 players (93%) reported 1439 injuries, of which 904 (63%) were NTLI and 535 (37%) were TLI. Most injured players had one NTLI (*n* = 136, 31%) or one TLI (*n* = 175, 40%) (Table 2). An average squad of 15 players can expect 49 injuries (31 NTLI and 18 TLI) per season.

**Table 2:** Injury frequency (NTLI and TLI) in the previous season, presented per player (*n*, %)

Players, NTLI *n* (%)	56 (13)	136 (31)	109 (25)	67 (15)	27 (6)	13 (3)	16 (4)	5 (1)	2 (1)	1 (0.2)	2 (0.8)	434 (100)
Injury numbers, *n*	0	1	2	3	4	5	6	7	8	9	10	Total
Players, TLI, *n* (%)	109 (25)	175 (40)	103 (24)	36 (8)	10 (2)	–	1 (1)	–	–	–	–	434 (100)

*NTLI* non-time-loss injury, *TLI* time-loss injury, *n* number

The most affected NTLI locations were the hip/groin (17%), knee (14%) and ankle (12%). The most affected TLI locations were the ankle (22%), knee (18%), hamstring (11%), thigh (10%), and hip/groin (9%) (Table 3). Of all 1439 injuries, 1261 (88%) were located in the lower body (including lumbar spine and pelvis).

**Table 3:** Injury location and ranking

Non-time-loss Injury (NTLI)			Time-loss Injury (TLI)		
Body location	Rank	*n* (%)	Body location	Rank	*n* (%)
Hip/Groin	1	154 (17)	Ankle	1	118 (22)
Knee	2	123 (14)	Knee	2	94 (18)
Ankle	3	110 (12)	Hamstring	3	57 (11)
Lumbar spine	4	92 (10)	Thigh	4	52 (10)
Thigh	5	83 (9)	Hip/groin	5	46 (9)
Hamstring	6	69 (8)	Lumbar spine	6	40 (8)
Calf	7	59 (7)	Calf	7	33 (6)
Foot	8	43 (5)	Foot	8	24 (4)
Shoulder	9	39 (5)	Head	9	14 (2)
Neck	10	30 (3)	Lower leg (front)	10	13 (2)
Lower leg (front)	11	29 (3)	Wrist/hand	11	12 (2)
Wrist/hand	12	22 (2)	Shoulder	12	10 (2)
Head	13	20 (2)	Pelvis	13	9 (1)
Pelvis	14	13 (1)	Trunk	14	6 (1)
Trunk	15	8 (1)	Neck	15	4 (1)
Elbow	16	6 (0.6)	Face	16	2 (0.8)
Face	17	4 (0.4)	Elbow	17	1 (0.2)
Total		904 (100)			535 (100)

Body location and ranking of non-time-loss (NTLI) and time-loss injuries (TLI) for all players (*n* = 434) in the previous season (n, %)

### Hip and groin injury during the previous season

For the previous season, 172 players (40%) reported 200 HGI. Of these 172 players, 28 players (6%) had both NTL-HGI and TL-HGI, 126 players (30%) sustained only NTL-HGI, and 18 players (4%) sustained only TL-HGI. The prevalence of NTL-HGI was 36% (154 injuries) and prevalence of TL-HGI was 11% (46 injuries) (Table 4). A history of HGI prior to the 2014–2015 season was reported in 166 players (38%). Of those, 101 players (23%) also sustained HGI in the 2014–2015 season.

**Table 4:** Prevalence of hip and groin injury

	Previous Season	Preseason
Players with HGI	172 (40)	117 (27)
Players with NTL-HGI	154 (36)	109 (25)
Duration		
Minor (1–7 days)	98 (22)	71 (16)
Moderate (8–28 days)	28 (7)	28 (7)
Major (> 28 days)	28 (7)	10 (2)
Players with TL-HGI	46 (11)	23 (5)
Duration		
Minor (1–7 days)	22 (5)	14 (3)
Moderate (8–28 days)	11 (2)	2 (1)
Major (> 28 days)	13 (3)	7 (2)

Self-reported prevalence of HGI (both non-time-loss and time-loss) in the previous season (2014–2015) and preseason of 2015–2016 (*n* = 434) is also reported for all duration groups. Data are presented as numbers (*n*) and rates (%)

*HGI* hip and groin injury, *NTL-HGI* non-time-loss hip and groin injury, *TL-HGI* time-loss hip and groin injury

The dominant leg was affected in 100 players (58%), and the non-dominant leg was affected in 33 players (19%); 39 players (23%) sustained bilateral HGI. The onset for HGI was maximal kicking (24%), sprinting/running (21%), pivoting/cutting (11%), and others (44%). An average amateur squad of 15 players can expect 5 NTL-HGIs and 2 TL-HGIs per season, resulting in 53 days of ongoing hip and groin problems and 21 days of play lost.

### Hip and groin injury during the preseason

During the preseason, 117 players (27%) reported 132 HGIs. Of these 117 players, 15 (3%) had both NTL-HGI and TL-HGI, 94 players (22%) sustained only NTL-HGI, and 8 players (2%) sustained only TL-HGI. The prevalence of NTL-HGI was 25% (109 injuries) and prevalence of TL-HGI was 5% (23 injuries) (Table 4). The dominant leg was affected in 60 players (51%), and the non-dominant leg was affected in 28 players (24%); 29 players (25%) sustained bilateral HGI.

### Severity of hip and groin injury

Players with HGI in the previous season had lower HAGOS scores in the preseason than players without HGI in the previous season (*p* < 0.001). Players with major HGI in the previous season had lower HAGOS scores in the preseason than those with minor HGI (*p* < 0.001) (Table 5).

**Table 5:** HAGOS subscale scores for players with hip and groin injury in the 2014–2015 season

HGI subgroups	Pain	Symptoms	ADL	SR	PA	QOL
No HGI (*n* = 262)	100.0 (97.5–100.0)	92.9 (85.7–100.0)	100.0 (100.0–100.0)	100.0 (100.0–100.0)	100.0 (75.0–100.0)	100.0 (100.0–100.0)
Difference HGI–No HGI	< 0.001	< 0.001	< 0.001	< 0.001	< 0.001	< 0.001
HGI (*n* = 172)	92.5 (80.0–97.5)	78.6 (71.4–89.3)	95.0 (80.0–100.0)	89.1 (74.3–100.0)	87.5 (75.0–100.0)	90.0 (75.0–100.0)
Difference no HGI–minor HGI	< 0.001	< 0.001	< 0.001	< 0.001	< 0.001	< 0.001
Minor HGI (*n* = 103)	95.0 (85.0–100.0)	82.1 (75.0–92.9)	100.0 (85.0–100.0)	93.8 (78.1–100.0)	87.5 (75.0–100.0)	95.0 (85.0–100.0)
Difference minor–moderate HGI	0.034	0.062	0.285	0.306	0.320	0.008
Moderate HGI (*n* = 30)	90.0 (77.5–95.0)	75.0 (67.9–89.3)	95.0 (80.0–100.0)	87.5 (71.9–100.0)	87.5 (75.0–100.0)	77.5 (70.0–95.0)
Difference moderate–major	0.078	0.283	0.170	0.046	0.012	0.016
Major HGI (*n* = 39)	77.5 (70.0–95.0)	75.0 (60.7–82.1)	90.0 (70.0–100.0)	75.0 (56.3–93.8)	75.0 (50.0–87.5)	70.0 (55.0–85.0)
Difference minor–major HGI	< 0.001	< 0.001	< 0.001	< 0.001	< 0.001	< 0.001

HAGOS scores (median, IQR) obtained at the current preseason for all players, for players with HGI, with no HGI and for the duration groups minor (1–7 days), moderate (8–28 days), and major (> 28 days) HGI, all in the previous season. *p* values are presented for differences between two subgroups

*HAGOS* hip and groin outcome score, *HGI* hip and groin injury, *ADL* activities of daily living, *SR* sport and recreational activities, *PA* participation in physical activity: *QOL* quality of life, *IQR* inter quartile range, *n* number

### Duration of hip and groin injury

**Fig. 2:**
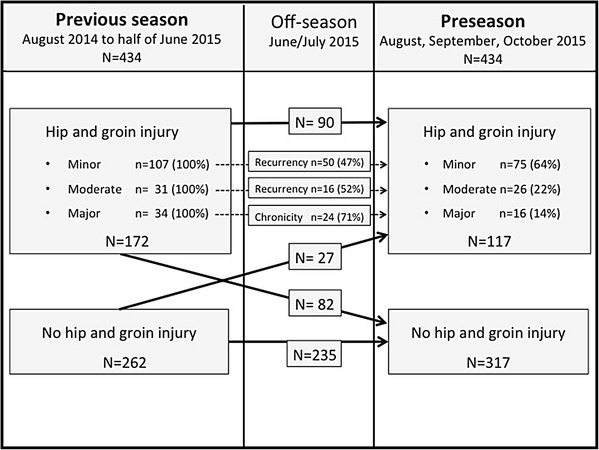
Player flow from the previous season to the current preseason for players with hip and groin injury per duration category, and for those with no HGI in the previous season

From the 172 players with HGI in the previous season, 82 (48%) had recovered and 90 (52%) sustained HGI in the following preseason. Of these, 66 (73%) were recurrent and 24 (27%) were chronic HGI. There were 50 recurrent HGI (47%) from the minor HGI group in the previous season and 16 (52%) from those with moderate HGI. The 24 chronic HGIs originated from the major HGI group (71%) in the previous season. Of the 117 HGIs in the preseason, 27 (23%) were new HGIs (Fig. 2; Table 4).

## Discussion

The most important finding of the present study was that hip and groin injury was the most prevalent non-time-loss injury in female amateur football players (17%). There was a high within-season prevalence of total injury (93%) and hip and groin injury (40%) and a high preseason prevalence of HGI (27%). Non-time-loss injuries were more prevalent than time-loss injuries. More than half of all hip and groin injuries in the previous season were recurrent or chronic injuries in the following preseason. The longer the duration of HGI in the previous season, the higher the chance of carrying over hip and groin problems into the following season.

### Presence of total injury

Non-time-loss injury rates (63%) were almost double that of time-loss injury rates (37%), which is in line with the previous studies in female collegiate sports [4, 17]. A TLI prevalence of 37% agrees with the previous studies in female football that used only a TLI definition (38–48%) [6, 11, 14, 20]. Of all injuries, 88% were located in the lower body, which was also found in the previous studies (82% [11], 87% [12], and 89% [20]). The hip/groin was the most frequently affected injury location (17%) for NTLI. TLI most often affected the ankle (22%) and knee (18%), which agrees with the previous reports in time-loss injury locations in elite female football [6, 11, 14, 20]. Non-time-loss injuries accounted for 63% and time-loss accounted for 37% of all injuries. Therefore, an average team of 15 players had 49 injuries (31 NTLI and 18 TLI) in the 2014–2015 season.

### Presence of hip and groin injury

Nearly half of the female amateur players (40%) sustained HGI in the previous season, which is similar to injury rates found in a Norwegian survey study in elite female players (45%) [9]. A Swedish survey study showed lower rates (28%) in sub-elite female players [27]. Seasonal incidences of 49% [26] and 55% [13] were found in male players. Female and male HGI incidence may be much more comparable than previously reported [30]. In this study, an average team had seven hip and groin injuries (five NTL-HGI and two TL-HGI) in one season, resulting in 53 days of ongoing hip and groin problems and 21 days of play lost.

In the previous season, 36% of all players continued playing despite hip and groin problems (NTL-HGI), whereas 11% had stopped playing for at least 1 day due to these problems (TL-HGI). Similar findings (36 vs. 9%) were recently shown in elite Norwegian female players [9]. The previous studies on female time-loss groin injury reported similar findings (2–11%) [5, 9, 14, 22], and a recent review reported that TL-HGI rates in males were twice as high as in females [30]. All these studies had more or less comparable exposure rates (148 in this study vs. 198 [14], 212 [5], and 213 [22] hours/player), yet a study with a higher exposure rate (393 h/player) also had a much higher injury rate (46%) [8]. It may be that injury rates depend more on exposure than on gender or playing level [1, 31, 33].

### Duration and severity of hip and groin injury

Half of the players (52%) with HGI in the previous season were still injured or re-injured after the off-season. This proportion was found to be one-third in male sub-elite players [24, 26]. In the new preseason, a quarter of all players (27%) reported hip and groin problems, with a full season still to come. This was also reported by male players, with a preseason prevalence of 36% [26]. As longstanding HGI related to more severe hip and groin problems (low HAGOS scores), not only a previous time-loss injury [34] but also the duration of hip and groin problems may relate to the risk of recurrence, chronicity, and time-loss [10, 26].

Players with longstanding HGI (> 1 month) had identical HAGOS scores on the subscales of pain and participation as those from a study in male players (> 1.5 months) [26].

### Clinical implications

Our study shows that there is a significant injury burden in female amateur football. Prevention of injuries has a high priority within the sport. We also found that how injuries are measured and defined affects the incidence rates, with TLI being only the tip of the injury iceberg. With regard to HGI, this study demonstrates the importance of a measurement tool to quantify not only time-loss yet also the severity of hip and groin problems for trainers, players, and medical staff. The results of this study showed that nearly half of the players with short-term HGI (< 1 week) sustained recurrent hip and groin injury during the following preseason. To identify players with increased risk for longstanding and severe hip and groin-related problems, regular assessment of hip and groin symptoms and sports performance should be performed [10, 26]. As the HAGOS has been developed and validated to measure symptoms and sports performance in detail, it is a useful tool for measuring severity of HGI instead of dichotomous reporting on time-loss injury [26].

This study used players from all KNVB districts across the whole country, instead of regional allocation that can possibly lead to allocation bias. To avoid underestimation of the actual injury burden of (overuse) injuries, both NTLI and TLI were assessed [1]. Players self-reported their injuries instead of medical staff, as many amateur clubs have no structured medical care. To increase the precision of reporting and target recall, we chose to use figures to specify anatomical regions.

We acknowledge a number of limitations. As this was a surveillance study without assessment by a medical professional, the classification of groin pain following the clinical entity approach, as recommended by the DOHA agreement [32], could not be performed. A correct diagnosis is mandatory for effective management and prognosis. Despite the type of questions used, recall bias may exist to some extent [29]. Retrospective, self-reported registration of the exact number of injuries, body region, and diagnosis may underestimate the prevalence of injuries, as minor injuries tend to be forgotten [18]. As the onset and recovery of injury were not registered, TL-injury numbers during a time-loss period could not be accounted for. Registration of the full length of training sessions and matches could have overestimated exposure. Players who responded at the beginning of the surveillance period had less time to become injured than players who responded at the end. Due to the retrospective study design, the influence of potential confounders could not be assessed. Further studies should consider the use of standardized clinical examination by medical professionals with a prospective design during a one-season period.

## Conclusion

Injury risk is high in female amateur football, with 93% of players sustaining an injury in a single season. Hip and groin injury is the most common non-time-loss injury and is three times more prevalent than time-loss HGI. Most players with longstanding HGI in the previous season still have residual hip and groin problems at the beginning of the new season.

## Electronic supplementary material

Below is the link to the electronic supplementary material.

Supplementary material 1 (DOCX 23 KB)

Supplementary material 2 (PDF 163 KB)
