# Supplementary material for: Hip and groin injury is the most common non-time-loss injury in female amateur football
Source: Knee Surg Sports Traumatol Arthrosc. 2018 Jun 2;27(10):3133–41. doi: 10.1007/s00167-018-4996-1 (PMC6754353; doi:10.1007/s00167-018-4996-1)
Supplement: Supplementary file 1 — Supplementary material 1 (DOCX 23 KB) [file 167_2018_4996_MOESM1_ESM.docx]

**Appendix (English version)**

**Questionnaire**

**Instruction**:

Please, answer all questions by ticking the appropriate box or formulating the answer as accurately as possible. Choose the answer option that most applies. The questionnaire consists of 2 parts and answering all questions will take 10 minutes. There is room for any comments or questions at the end of the questionnaire.

**Question 1**. What is your age?

**Question 2.** What is your weight (rounded up to full kg)?

**Question 3.** What is your length (rounded to full cm)?

**Question 4**. At what level did you play during the 2014-2015 season (field football only)? Top Class, Sub-Top Class, 1st Class, 2nd Class, 3rd Class, 4th Class, 5th Class, 6th Class

**Question 5.** How many matches did you play in the last season (2014-2015)? (field football only)?

**Question 6.** How many hours did you train last season on average per week (season 2014-2015) (field football only)? ...... hour

**Question 7.** What is the preferred leg to kick with (one answer)?: Left / Right

**Question 8.** Did you sign up as part of your team or as individual?

**QUESTIONS CONCERNING THE PREVIOUS SEASON 2014-2015, FIELD FOOTBALL:**

The following questions relate to the previous season (2014-2015) from the period 01-08-2014 to 15-06-2015.

**Definition injury:** *Any physical complaint sustained as a result of a football match or training, resulting in being unable to fully take part in future football training or match play (time loss)*

**Question 9:**

Have you had hip and groin injury in the period prior to the start of the previous season 2014-2015 (31-07-2015)?

□ Yes.

□ No.

□ I do not know

**Question 10**:

Please indicate per body location (Figure 1), the injuries you had during the 2014-2015 season (01-08-2014 to 15-06-2015).

- Head, face, neck / neck, shoulder, elbow, wrist / hand, skirt, hip / groin, upper leg, knee, lower leg for ankle / foot, back low, pelvis, upper leg back, calf / achilles tendon, no injury .

**Definition complaint:** *Any physical complaints that are bothering you, but who do not prevent you from playing field football or being unable to play the next field match (s) or field training (s) (non-time loss).*

**Question 11:**

Please indicate per body location (Figure 1), the area where you have had complaints during the 2014-2015 season (01-08-2014 to 15-06-2015).

- Head, face, neck / neck, shoulder, elbow, wrist / hand, skirt, hip / groin, upper leg, knee, lower leg for ankle / foot, back low, pelvis, upper leg back, calf / achilles tendon, no obstacle.

**Definition hip and groin complaints:** *Physical complaints in the region between the front of the hip and the inside of the upper leg that are bothering you, but who do not prevent you from playing field football or being unable to play the next field match (s) or field training (s) (non-time loss).*

**Question 12:**

What kind of hip and groin complaints (one or more) did you suffer during the 2014-2015 season (01-08-2014 to 15-06-2015):

□ Symptoms of hip groin region: feeling of discomfort, clicks or other sounds, bending sideways outwards, difficulty in taking full fit, sudden stabbing or shoots.

□ Stiffness in hip groin region in the morning or later in the day.

□ Hip groin pain in a region that you think is related to hip and groin complaints

□ Pain during bending/stretching of the hip/groin, stairwalking, sleeping, lying, sitting, standing, walking (on hard or uneven ground)

□ Restrictions in daily life in activities such as stairwalking upwards, bending, getting in/out of cars, lying in bed/turning, household work

□ Restrictions in sports practice by hip and groin complaints: squatting, running, turning/walking, walking on uneven surfaces, sprinting, shooting.

□ I do not know

□ Not applicable: No complaints.

**Question 13:**

How long have you experienced hip and groin complaints during the 2014-2015 season (01-08-2014 to 15-06-2015):

□ Because of my hip pains I did not play football.

□ I had 1-7 days hip/groin complaints, but I have been able to play football.

□ I had 8-28 days hip/groin complaints, but I have been able to play football.

□ I had more than 28 days hip/groin complaints, but I have been able to play football.

□ I do not know.

□ Not applicable: No complaints

**Definition** **hip and groin injury***: Physical complaints in the region between the front of the hip and the inside of the upper leg, sustained as a result of a football ball match or training, resulting in being unable to fully take part in future football training or match play (time loss)*

**Question 14:**

How many days have you not been able to play football due to this particular hip and groin injury in the 2014-2015 season?

□ Despite the complaints, I have just been able to play football.

□ I could not play football for 1-7 days due to my hip/groin complaints.

□ I could not play football for 8-28 days due to my hip/groin complaints.

□ I could not play football for more than 28 days due to my hip/groin complaints.

□ I do not know.

□ Not applicable: No complaints.

**Question 15:**

Which side was affected by hip and groin complaints during the 2014-2015 season?

□ Links.

□ Right.

□ I do not know.

□ Not applicable: No complaints.

**QUESTIONS CONCERNING THE CURRENT PRESEASON 2015-2016, FIELD FOOTBALL:**

The following questions relate to the current preseason (2015-2016) from the period 01-08-2014 to 04-11-2016.

**Definition hip and groin complaints***: Physical complaints in the region between the front of the hip and the inside of the upper leg that are bothering you, but who do not prevent you from playing field football or being unable to play the next field match (s) or field training (s) (non-time loss).*

**Question 16:**

How long have you experienced hip and groin complaints in the period from 01-08-2015 to 04-11-2015?:

□ Because of my hip/groin complaints I can not play football.

□ I have 1-7 days hip/groin complaints, I can play football.

□ I have 8-28 days hip/groin complaints, I can play football.

□ I have more than 28 days hip/groin complaints, I can play football.

□ I do not know

□ Not applicable, no complaints.

□ Not applicable, I am currently not playable for reasons other than hip and nose complaints

**Question 17:**

From what football activities do you experience your current hip and groin complaints (between 01-08-2015 and 04-11-2015, multiple answers possible):

□ Because of my hip and groin complaints I can not play football.

□ Maximum (at your hardest) kicking a ball.

□ jogging

□ Sprinting.

□ Turn and turn.

□ Otherwise; ...... ..

**Question 18:**

When do you expierience your current hip and groin complaints (between 01-08-2015 and 04-11-2015, multiple answers are possible):

□ Only after field football.

□ At the start of field football, but not during field football.

□ At start, during and after field football.

□ At start-up, during and after field football, with performance reduction due to my hip and groin complaints.

□ Otherwise: .......

**Definition hip and groin *injury****: Physical complaints in the region between the front of the hip and the inside of the upper leg, sustained as a result of a football match or training, resulting in being unable to fully take part in future football training or match play (time loss)*

**Question 19:**

How many days have you not been able to play football due to your hip and groin injury in the current season (01-08-2015 to 04-11-2015)?

□ Despite the complaints, I have just been able to play football.

□ I could not play football for 1-7 days due to my hip/groin complaints.

□ I could not play football for 8-28 days due to my hip/groin complaints.

□ I could not play football for more than 28 days due to my hip/groin complaints.

□ I do not know.

□ Not applicable, no complaints.

□ Not applicable, I am currently not playable for reasons other than hip and groin complaints.
