# Supplementary material for: Hip and groin injury is the most common non-time-loss injury in female amateur football
Source: Knee Surg Sports Traumatol Arthrosc. 2018 Jun 2;27(10):3133–41. doi: 10.1007/s00167-018-4996-1 (PMC6754353; doi:10.1007/s00167-018-4996-1)
Supplement: Supplementary file 2 — Supplementary material 2 (PDF 163 KB) [file 167_2018_4996_MOESM2_ESM.pdf]

This study complied with the requirements of the declaration of Helsinki[24]. The Dutch Central Committee on Research Involving Human Subjects (CCMO) states that no medical ethical approval was necessary for this questionnaire study, as stated in the Dutch Medical Research Involving Human Subjects Act (http://www.ccmo.nl/nl/toetsingscommissie-ccmo-of-metc?55a37b93-dd8c-4bf8-8883-2d30c35ff8ba).

By clicking the “I participate” link in the electronic questionnaire, participants gave their consent that their anonymized data would be used for research purposes.

Help-me-on-my-way!

This instrument assists you in the reviewing procedure for medical/scientific research. If the equipment you use for visiting this site does not allow you to use this tool please visit the static version of this tool.

Does my research have to undergo a medical ethical review?

First you must determine if your research falls under the scope of the WMO. The window on the side with the title 'WMO' explains what this means.

My research falls under the WMO

Does your research fall under one of the areas given on the side window?

☐ *My research falls under one of these areas*

☑ *My research does not fall under one of these areas*

My research does not fall under the WMO

I'm not sure

Depending on the field of research your research proposal must be reviewed by either the CCMO or an accredited MREC.

Does it concern research with/in the field of:

- a non-authorised vaccine
- antisense oligonucleotides
- interference-RNA
- (somatic) cell therapy
- gene therapy
- genetically modified organisms (GMOs)
- gametes or (leftover) embryos (with the exception of non-invasive observational research on the foetus)
- xenotransplantation of live cells from animals
- Research with products that fall under the Opium Act in the case of treatment for addiction to these products.

The types of research given above must undergo a central review by the CCMO. For more information see the Central Review Decree (Besluit Centrale Beoordeling, BCB, in Dutch) and the Amendment Decree of the Central Review Decree (Besluit tot wijziging van Besluit Centrale Beoordeling, in Dutch).
